# Mining gene networks with application to GAW15 Problem 1

**DOI:** 10.1186/1753-6561-1-s1-s52

**Published:** 2007-12-18

**Authors:** Jing Hua Zhao, Jian'an Luan, M Fazil Baksh, Qihua Tan

**Affiliations:** 1 MRC Epidemiology Unit, Institute of Metabolic Science, Box 285, Addenbrooke's Hospital, Hills Road, Cambridge CB2 0QQ, UK; 2 Section of Applied Statistics, School of Biological Sciences, The University of Reading, Earley Gate, Reading RG6 6FN, UK; 3 Department of Biochemistry, Pharmacology and Genetics, Odense University Hospital, Sdr Boulevard 29, Odense C., DK-5000, Denmark

## Abstract

The Genetic Analysis Workshop 15 (GAW15) Problem 1 contained baseline expression levels of 8793 genes in immortalized B cells from 194 individuals in 14 Centre d'Etude du Polymorphisme Humain (CEPH) Utah pedigrees. Previous analysis of the data showed linkage and association and evidence of substantial individual variations. In particular, correlation was examined on expression levels of 31 genes and 25 target genes corresponding to two master regulatory regions. In this analysis, we apply Bayesian network analysis to gain further insight into these findings. We identify strong dependences and therefore provide additional insight into the underlying relationships between the genes involved. More generally, the approach is expected to be applicable for integrated analysis of genes on biological pathways.

## Background

Recent genetic dissection of common diseases has largely been through linkage and association studies involving discrete or continuous traits including intermediate phenotypes such as gene expression data from microarray experiments. The latter can involve thousands of genes, and annotation of their roles in biological pathways and in relation to DNA polymorphisms poses immense challenges and has sparked huge interest [1]. These include development of methods appropriate for a much richer structure than classic clustering [2], discovery of interaction between genes, and inference of causal relationships.

A key challenge in analysis of gene expression data is the reconstruction of regulatory networks. Several approaches directly extend classical techniques such as cluster analysis to infer the relationship between plural variables. A novel but apparently unpopular approach of cluster analysis is to extract the patterned information formally and use it in typical linkage and association analyses. More importantly, cluster analysis can be followed by Gaussian graphical modelling [2,3] and multivariate analysis in which a partial correlation coefficient (instead of a correlation coefficient) is used to measure the direct interaction between variables. In graphical modelling, the relationship between plural variables is represented as an independence graph G = (V, E), whose vertices V denote variables and edges E denote conditional dependence structure. Other approaches include regularization and moderation for suitable estimates of the covariance matrix and its inverse, by a full Bayesian or an empirical Bayes approach and followed by heuristic searches for an optimal graphical model . A Bayesian network is notable because it provides a natural approach to model regulatory networks. As has been argued elsewhere [4], if the expression level of a given gene is regulated by certain proteins then it should be a function of the active levels of these proteins. Due to biological variability and measurement errors, the function would be stochastic rather than deterministic. A Bayesian network uses a generic analytic approach for identifying robust predictors of among-individual variation in expression levels, intermediate phenotypes, or disease end points. It has been successfully applied to *APOE *gene variation and plasma lipid levels [5]. Mathematical details on Bayesian networks are available [6], as is a comprehensive survey of genomic approaches to biological pathways [7].

The Problem 1 data from Genetic Analysis Workshop 15 (GAW15) offers an excellent opportunity for investigating the utility of Bayesian networks. An earlier report [8] showed evidence of substantial variation in expression levels between individuals and association with single-nucleotide polymorphisms (SNPs), as well as a cluster of 25 of 31 target genes in two master regulatory regions. Here, as a further step of analysis, we performed Bayesian network modelling to gain insight into these findings.

## Methods

Gene expression levels, treated as continuous variables, can be assumed to follow a multivariate normal distribution, and to be consistent with a Bayesian network with linear Gaussian conditional densities. The prior of this network is characterized by a prior network reflecting our belief in the joint distribution of the variables in question, and equivalent sample size (ESS) effectively behaving as if it was calculated from a "prior" data set of that size. For instance, without *a priori *knowledge of the regulatory network, the prior network could be one in which all expression levels are independent in order to avoid explicitly biasing the learning procedure to a particular edge. The common approach to the learning procedure starts with a training set and evaluates networks according to an asymptotically consistent scoring function that is obtained through the Bayesian framework [6]. In the case of B-course software  to be used here, discretisation of continuous data has been applied to capture the nonlinear relationship between variables and the choice of prior is such that the resulting ESS prior distribution is close to Jeffrey's prior. The software infers causal relationship according to the statistical dependence under some additional assumptions concerning latent variables. Mathematical details, including the definition of Jeffrey's prior, are given elsewhere [9].

The GAW15 Problem 1 consists of 194 individuals from 14 three-generation CEPH (Centre d'Etude du Polymorphisme Humain) pedigrees, with baseline expression levels of genes in immortalized B-cells. The data provided contains expression of 8793 genes. Following an earlier investigation [8], expressions whose variations are greater among individuals than within individuals are considered, leading to 3554 expressions. By further considering the evidence of master regulations, mapping was done without taking into account possible relationships among phenotypes, leading to 25 of the 31 target genes. These were used here for the network analysis, involving 56 unrelated individuals.

Affymetrix CEL-files were preprocessed with BioConductor package *affy*, but the target gene expressions were used directly. The probe set IDs were matched with the annotation database of human genome focus array distributed with GAW15 Problem 1 and from the Affymetrix website . All data management, correlation and hierarchical cluster analysis were done with the R system .

## Results

**Figure 1:**
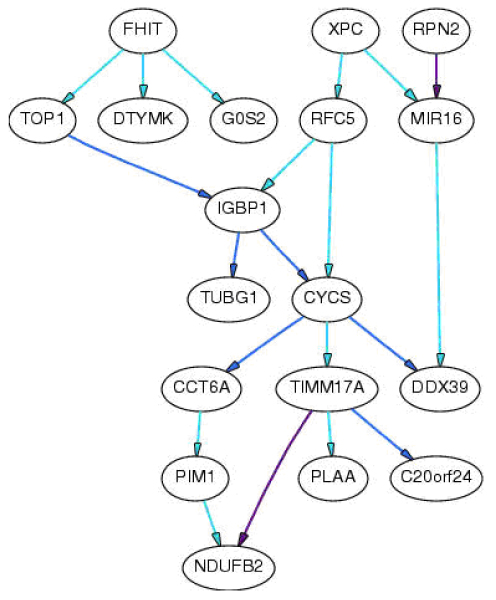
**Importance of the dependencies**. Solid line has direct causal influence ("direct" means that causal influence is not mediated by any other variable that is included in the study).

**Figure 2:**
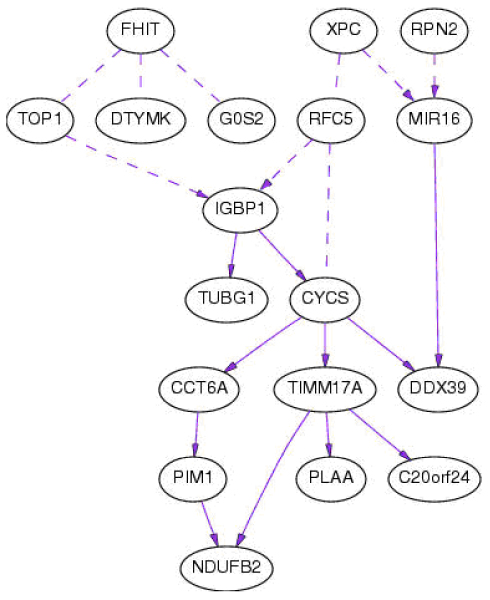
**Importance of the causal structure**. Solid line has direct causal influence ("direct" means that causal influence is not mediated by any other variable that is included in the study). Dashed line indicates there are two possibilities, but we do not know which holds. Dashed line without any arrow heads indicates there is a dependency but we do not know the reciprocal dependence.

Cluster analysis shows that the dendrogram (not shown) differs somewhat from the earlier report [8], possibly due to difference in sample sizes. Network analysis using B-course (100^th ^checkpoint) showed that the following genes are independent of any other genes in the model: *NFYC*, *LSM3*, *RAN*, *VAMP2*, *RAP80*, *INPP5A*, *STC2*, and *SNRPB*. Edges *TIMM17A *to *NDUFB2 *and *RPN2 *to *MIR16 *are very strong and removing any of them would result in a model with probability less than one millionth that of the original model. Other results are shown in Table 1. Removing any of the edges in Edge Set 1 from the chosen model would decrease the probability of the model to less than one thousandth the probability of the original model, while removing any of the edges in Edge Set 2 decreases the probability of the model by the ratio listed. The network models are shown in Figures 1 and 2. The so-called causal structure assumes that dependencies between variables are due to causal relationships between variables in the model.

**Table 1:** Strength of the dependency. Removing any of the edges (Vertex 1 to Vertex 2) in edge set one from the chosen model would decrease the probability of the model to less than one thousandth the probability of the original model, while removing any of the edges in edge set two decreases the probability of the model (exact ratio listed).

Edge Set 1			Edge Set 2		
	
Vertex 1	Vertex 2	Ratio	Vertex 1	Vertex 2	Ratio
TOP1	IGBP1	436736	CYCS	TIMM17A	102
TIMM17A	C20orf24	201449	RFC5	CYCS	92
CYCS	CCT6A	89880	FHIT	TOP1	61
IGBP1	TUBG1	16221	PIM1	NDUFB2	41
CYCS	DDX39	9248	RFC5	IGBP1	31
IGBP1	CYCS	4388	FHIT	DTYMK	17
			XPC	MIR16	15
			XPC	RFC5	9.96
			FHIT	G0S2	5.85
			MIR16	DDX39	3.58
			TIMM17A	PLAA	3.57
			CCT6A	PIM1	3.31

## Discussion

Our analysis provides new insights into the complex interactions of gene expression levels in GAW15 Problem 1 data. This work demonstrates the potential usefulness of statistical inference on causal structure. Without an *a priori *biological hypothesis, it serves as an exploratory tool for subsequent confirmatory analysis. We chose not to repeat the linkage and association analysis but use earlier findings directly [8] and have used the non-informative prior in the analysis as in the current version of B-course. More generally, the influence of the prior network can depend on a variety of factors and is the subject of ongoing research.

An apparent limitation of this work, though not uncommon in gene-expression studies, is the relatively small sample size used. To fully elucidate the biological pathways involved may be difficult. For example, *CYCS *is involved in six pathways according to . Nevertheless, this would be a useful step towards understanding of the biological mechanism underlying the master regulators in question. A further limitation relates to the assumption often made in analysis of gene expression data that expression levels of genes are proxies for the activity level of the proteins they encode, although there are numerous examples in which activation or silencing of a regulator is carried out by post-transcriptional protein modifications. Statistical robustness and biological interpretability remain as the two main challenges for Bayesian network analyses, to which replication, bootstrapping and benchmarking have been proposed.

Our inference of gene networks also exploits the covariance structure of the data, like structural equation modelling [10,11], but it is exploratory or hypothesis-generating rather than confirmatory or hypothesis-driven. A number of other software systems are of interest, e.g., ASIAN (a web-based regulatory network framework [12], ) and deal [13]. The B-course software can also generate input files for HUGIN, a commercial tool for inference with Bayesian networks . Further investigations would be fruitful and may involve genotype data, comparison between groups [5], or SNPs within the same gene, among others.

## Conclusion

Bayesian network modelling is applied to GAW15 gene expression data and shown to be more informative than classic cluster analysis. While the findings are the subject of further investigation, the approach merits further attention.

## Competing interests

The author(s) declare that they have no competing interests.
